# WinPCA: a package for windowed principal component analysis

**DOI:** 10.1093/bioinformatics/btaf529

**Published:** 2025-09-22

**Authors:** L Moritz Blumer, Jeffrey M Good, Richard Durbin

**Affiliations:** Department of Genetics, University of Cambridge, Cambridge, CB2 3EH, United Kingdom; Division of Biological Sciences, University of Montana, Missoula, MT 5981, United States; Division of Biological Sciences, University of Montana, Missoula, MT 5981, United States; Department of Genetics, University of Cambridge, Cambridge, CB2 3EH, United Kingdom

## Abstract

**Summary:**

With chromosomal reference genomes and population-scale whole genome-sequencing becoming increasingly accessible, contemporary studies often include characterizations of the genomic landscape as it varies along chromosomes, commonly termed genome scans. While traditional summary statistics like *F*_ST_ and *d*_XY_ between pre-assigned populations remain integral to characterizing the genomic divergence profile, PCA differs by providing single-sample resolution, thereby supporting the identification of polymorphic inversions, introgression and other types of divergent sequence that may not be fully aligned with global population structure. Here, we introduce WinPCA, a user-friendly package to compute, polarize and visualize genetic principal components in windows along the genome. To accommodate low-coverage whole genome-sequencing datasets, WinPCA can optionally make use of PCAngsd methods to compute principal components in a genotype likelihood framework. WinPCA accepts variant data in either VCF or BEAGLE format and can generate rich plots for interactive data exploration and downstream presentation.

**Availability and implementation:**

WinPCA is implemented in Python and freely available at https://github.com/MoritzBlumer/winpca and https://doi.org/10.5281/zenodo.15614979.

## 1 Introduction

The combined actions of selection, gene flow, drift, and recombination shape the genomic landscape of populations, causing differences in local genetic composition along chromosomes (Seehausen *et al.* 2014). Principal component analysis (PCA) has become solidly established in population genetics since initial applications to large-scale human datasets alongside the publication of the EIGENSOFT software (Price *et al.* 2006) and is a cornerstone of genetic variation studies today. Here, we present WinPCA, a software tool to compute and visualize genetic principal components from biallelic whole genome sequencing (WGS) datasets in windows along chromosomes. WinPCA was inspired by Li and Ralph (2019) and more specifically Jay *et al.* (2021), who, to our knowledge, first introduced the idea to directly visualize polarized principal components along the genome to explore local variation in genetic structure. Versions of WinPCA have been used to detect chromosomal inversions (Blumer *et al.* 2025) and to characterize the mosaic structure of a mouse hybrid’s genome (Quiroga-Carmona *et al.* 2025). WinPCA addresses two of the key challenges frequently encountered with windowed PCA, (i) the treatment of missing data and (ii) the polarization of principal components along chromosomes. Since PCA algorithms inherently require complete datasets (Dray and Josse 2015), variants with any missing genotype calls are typically discarded from the input. Especially with large sample datasets this can quickly result in the exclusion of most variants. WinPCA by default mean-imputes missing genotype calls like other popular softwares. Alternatively, avoiding genotype imputation and at the same time accommodating low coverage WGS datasets, WinPCA can operate in a genotype likelihood framework by internally using PCAngsd (Meisner and Albrechtsen 2018) machinery. WinPCA scales to large datasets—we have applied it to whole genome variant callsets of thousands of samples.

## 2 Methods

WinPCA is written in Python and designed to be run from the UNIX command line. It is structured in a modular way, partitioning the windowed PC analysis into multiple steps from the computation of principal components to the creation of interactive output plots. Subcommands are called by typing “winpca *subcommand*”. The minimal input is a variant file in VCF, TSV, or BEAGLE (as used by the ANGSD suite (Korneliussen *et al.* 2014)) format. Below, we provide an overview of the five modules.

### 2.1 winpca pca

The core module, *pca*, parses variant files containing either hard-called genotypes or genotype likelihoods. Input files may be gzip- or bgzip-compressed (Li 2011). Biallelic variants are parsed in a rolling window fashion and a principal component analysis is conducted per window using either scikit-allel (Miles *et al.* 2025) for hard-called genotypes or PCAngsd (Meisner and Albrechtsen 2018) for genotype likelihoods (GL or PL format). Particularly for low coverage WGS datasets or datasets with large numbers of samples, for which hard genotype callsets frequently contain at least one missing genotype at a large fraction of sites, we recommend supplying properly imputed genotype call sets or genotype likelihoods as input to WinPCA [see Loh *et al.* (2016) and Davies *et al.* (2016) for reference panel-free genotype imputation methods]. When this is not done, WinPCA imputes missing genotype calls as the mean value on the fly. Despite limitations, this is the default for other commonly used genetic PCA algorithms [e.g. PLINK Purcell *et al.* (2007)], and is arguably preferable to discarding large amounts of information. Rolling windows are sequentially processed to limit memory requirements, and eigenvectors for the specified principal components are computed (by default PC1 and PC2), alongside metadata, per window. The results are then written to a set of gzipped text files that can be used by subsequent modules.

### 2.2 winpca polarize and winpca flip

Since PCA is performed separately for each genomic window, the polarity of a given PC is inhomogeneous across the output. To reduce the resulting noise for visualization purposes, WinPCA *polarize* attempts to harmonize the sign polarity of eigenvectors across adjacent windows. By default it does this by adaptively selecting the sample with the highest absolute PC value and using the sign average of the five previous (and already polarized) windows to decide whether to flip the current window. We call this adaptive auto-selection.

However, this does not always give the desired result, so we provide a variety of alternatives. First, it is possible to change the number of windows considered away from five. Alternatively polarization can be determined by the user specifying one or more fixed guide samples. For example where a natural outgroup is contained in the sample set, it makes sense for one or a group of samples to remain consistently positive or negative in PC space. While polarization by default is performed as part of the *pca* module, output data from an existing *pca* run may be re-polarized by invoking the *polarize* module directly, updating the output files. The *flip* module allows to reflect PCs for an entire chromosome to homogenize their orientation across the genome and furthermore accepts a list of chromosomal coordinates to flip individual windows if incorrectly polarized regions remain after automatic polarization.

### 2.3 winpca chromplot and winpca genomeplot

The plotting modules *chromplot* and *genomeplot* generate interactive plots which may be annotated with information from a user-provided metadata file. The plotly library (https://plotly.com/python) is used internally, which can generate a variety of output formats. Static plots may also be exported in vector (PDF, SVG) or raster formats (PNG), while HTML output files can be viewed interactively in a web browser. In this case metadata annotations are displayed when hovering over data points and one may zoom in and out of the plotting area or hide and view data groups. This allows for great flexibility in data exploration, while reducing the need for repeated plot generation at different scales or with alternative annotations. While *chromplot* visualizes a PC along a single chromosome, *genomeplot* jointly visualizes data across multiple chromosomes. Plotting colors, plotting order and plot interval can be controlled by the user. Quantitative metadata (e.g. phenotype scores) can be supplied to color-code samples using a continuous spectrum, which can help visualize quantitative trait loci (QTL).

## 3 Results

We illustrate the functionality and versatility of WinPCA analyses using four publicly available datasets. These vary in taxonomic group, number of samples, sequencing depth (called genotypes versus genotype likelihoods) and divergence level. Table 1 summarizes the key characteristics of each dataset. All analyses were conducted with the default window size of 1 Mbp if not stated otherwise.

**Table 1.** Key characteristics of the analyzed datasets.^a^

Dataset	No. samples	Variant type	File format	Missingness	No. variants (chrom)	Window (step) size	No. windows	Runtime
Humans	2 504	GT	VCF	Imputed	5 013 617 (chr1)	1 Mbp (100 kb)	2 480	23 h 09 min
Cannabis	110	GT	VCF	Unimputed	1 391 697 (chr1)	1 Mbp (10 kb)	10 399	34 min
Cichlid cross	290	GT	VCF	Unimputed	297 152 (chr7)	1 Mbp (10 kb)	6 740	2 h 55 min
Mouse hybrid	71	GL	BEAGLE	-	4 070 751 (chr1)	1 Mbp (25 kb)	8 213	2 h 30 min

a For each dataset the number of samples, variant type and file format are given. Additionally, the elapsed run time for the largest chromosome and major factors determining run time (number of variants, window size and step size) are provided.

**Figure 1.**
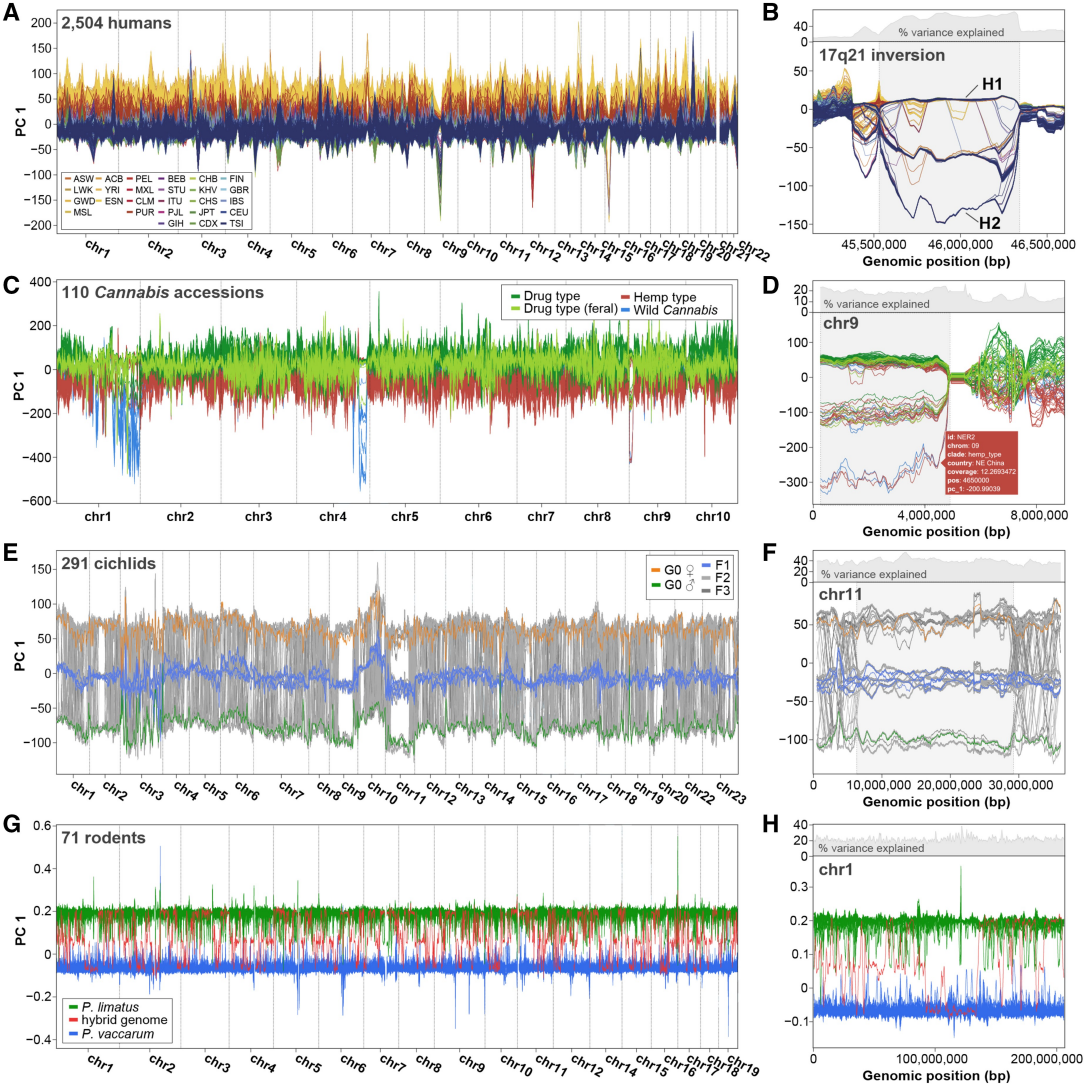
WinPCA use cases. We applied WinPCA to four exemplary datasets. Each sample is represented as a single line in WinPCA output plots. (A, B) 2504 humans from the 1000 Genomes Project color-coded by population. (B) Inversion polymorphism at 17q21 (highlighted in grey) at increased resolution with H1 and H2 haplotypes annotated. (C, D) 110 whole genome-sequenced *Cannabis* accessions including wild *Cannabis*, hemp-type and drug-type strains. (D) Polymorphic inversion spanning approximately 0–5 Mbp (highlighted in grey) on chromosome 9 at increased resolution. For reference, metadata displayed when hovering over a trace in the interactive HTML version of this plot is displayed. (E, F) 290 cichlid genomes from an interspecific cross (up to generation F3) between *Aulonocara stuartgranti* and *Astatotilapia calliptera*. (F) Separate display of chromosome 11. While F1 individuals localize intermediate between the parents in PC space, individual recombinations can be traced in F2 and F3 individuals. Full recombination suppression is evident in the inversion region (highlighted in grey). (G, H) Genomes from 70 *Phyllotis vaccarum* and *P. limatus* individuals and that of a single hybrid individual. The mosaic ancestry of the hybrid suggests an early but advanced stage of hybridization between both species (see text).

In the first example we used WinPCA to visualize the divergence landscape across 26 human populations (2504 individuals) that were sequenced for the 1000 Genomes Project (Auton 2015) (Fig. 1A). We then focused on the well-studied 17q21 inversion region with an increased resolution (100 kb window size) local scan (Fig. 1B). The core inversion spans 900 kb, is polymorphic in many human populations with elevated frequencies in some European groups and the derived H2 haplotype has been associated with multiple disease phenotypes (Campoy *et al.* 2022). The visible tripartite stratification into the three possible haplotype combinations is characteristic for PCA in inversion-polymorphic genomic regions (Ma and Amos 2012).

We next used WinPCA to analyze a dataset of 110 whole genome-sequenced *Cannabis sativa* accessions representing the global diversity of wild and cultivated lineages (Ren *et al.* 2021). In several regions on chromosomes 1 and 4 most wild lineages diverge from hemp and drug-type strains that share a history of cultivation (Fig. 1C). These regions overlap with previously identified genomic segments of increased *F*_ST_ (Woods *et al.* 2023). We noticed an additional highly divergent region on chromosome 9 and performed an increased-resolution (500 kb window size) local scan of this region (Fig. 1D). For reference, we included a hover display item from the HTML version of this plot. The stratified segregation pattern at approx. 0 to 5 Mbp suggests the presence of a megabase-scale inversion polymorphism that segregates among all four clades but went undetected in previous *F*  _ST_ scans. Interestingly, among the included samples, only hemp-type and wild *Cannabis* representatives carry the putative homozygous inverted genotype and the region is overrepresented among positively selected genes identified in a separate dataset (Woods *et al.* 2023, Supplementary Table 4). Some of these genes relate to traits relevant in plant environmental adaptation and cultivation [e.g. WRKY26 has a function in thermotolerance (Li *et al.* 2011) and UVR8 is a photoreceptor that modulates the response to ultraviolet radiation (Liu *et al.* 2024)]. A recent preprint reports this inversion in an alignment-based approach, thereby confirming its presence (Lynch *et al.* 2025).

Third, we include a dataset from our recent study of large chromosomal inversion polymorphisms in Lake Malawi haplochromine cichlids (Blumer *et al.* 2025). Figure 1E features 290 individuals from an interspecific cross up to generation F3 between *Aulonocara stuartgranti* and *Astatotilapia calliptera*. Individuals of the F1 generation combine one copy of each parental genome and consistently localize intermediate in PC space. In contrast, individuals from subsequent generations that underwent recombinations alternate between tracts that are homozygous for either parent’s ancestry or heterozygous. Individual recombination events are distinguishable at higher resolution (Fig. 1F) and regions of recombination suppression, especially inversions on chromosomes 2, 9, 11, and 20 (Blumer *et al.* 2025) can be easily spotted (Fig. 1E and F).

Finally, we analyzed a low (median: 2.58X) coverage dataset comprising 71 genomes of leaf-eared mice (*Phyllotis*) that is featured in our recent study (Quiroga-Carmona *et al.* 2025). WinPCA was used to visualize the mosaic ancestry of an individual from a *P. vaccarum—P. limatus* hybrid zone (Fig. 1G and H). The hybrid genome alternates between three types of local ancestries, comparable to the previously described cichlid cross. The presence of multiple short tracts with homozygous ancestry for each parental species suggests that the hybrid individual is at an advanced stage of intercross rather than a first generation hybrid. Given the approximately equal ancestry contributions from both *P. vaccarum* and *P. limatus*, the presence of a hybrid population could be inferred, although only a single specimen from that population has been sequenced (Quiroga-Carmona *et al.* 2025).

## 4 Discussion

Windowed principal component analysis represents a means to examine the genomic divergence landscape as it varies from genome-wide population structure, and allows to characterize datasets at single-sample resolution.

While for *F*_ST_ -like approaches genome-wide assignment of samples to populations can mask locally increased divergence patterns if they segregate across populations (as inversions frequently do) or are limited to only a few samples in a population, windowed PCA can often overcome these limitations. However, of course it has its own limitations. Principal component analysis is a dimensionality reduction method and the relevant axes of variation may be distributed across several of the higher order principal components (Berner 2011). WinPCA calculates PC1 and PC2 by default, but allows output of other PCs up to PC10. We have observed that known inversion polymorphisms are usually represented in PC1, but this might not always be the case and depends on the structure of the dataset. Due to the effect of noise in genomic variant data we recommend relatively large window sizes for genome-wide scans (100 kbp to 1 Mbp; the optimal value will depend on SNP density and linkage disequilibrium amongst other factors), and emphasize the benefits of high quality reference genomes with a low degree of fragmentation and misassembly. While WinPCA can parse genotype likelihoods from any source, we have observed good results with likelihoods produced by ANGSD with its standard defaults (Korneliussen *et al.* 2014).

WinPCA focuses on visualizing changes in local genetic structure along the genome, rather than analyzing the variation in this structure across the genome as in lostruct (Li and Ralph 2019). It should be noted that while WinPCA can be useful to identify local changes in genetic structure that are characteristic of inversions (see Fig. 1B, D, and F) other combinations of two divergent segregating haplotypes occurring in homozygous and heterozygous states can create a tripartite pattern. Furthermore, in the case of large inversions, recombination suppression can extend beyond the physical inversion boundaries (Li *et al.* 2023). We also note that there are other sources of outliers in local genomic structure, such as selection, that may be better identified through single variant tests such as pcadapt (Luu *et al.* 2017).

We provide WinPCA as an easy-to-use, scalable tool for initial unbiased exploration of WGS datasets (low or high coverage) and as a powerful method to identify and visualize divergent genomic regions reflecting various underlying genetic causes, as illustrated in the described examples. The automated classification of these divergence patterns is a separate task.

## Data Availability

All genetic datasets used are available from public databases. 2504 human genomes: http://ftp.1000genomes.ebi.ac.uk/vol1/ftp/data_collections/1000G_2504_high_coverage/working/20220422_3202_phased_SNV_INDEL_SV/; 110 Cannabis genomes: doi.org/10.5061/dryad.9p8cz8wfr; 290 cichlid genomes: https://doi.org/10.6084/m9.figshare.30136306; 71 rodent genomes: https://doi.org/10.6084/m9.figshare.30138433. Metadata used to annotate plots were derived from the associated publications and are available from https://github.com/MoritzBlumer/misc/raw/refs/heads/main/aux.zip.
